# Evaluation of the Effect of a Safe Medication Strategy on Potentially Inappropriate Medications, Polypharmacy and Anticholinergic Burden for People with Dementia: An Intervention Study

**DOI:** 10.3390/healthcare11202771

**Published:** 2023-10-19

**Authors:** Ashley Kable, Samantha Fraser, Anne Fullerton, Carolyn Hullick, Kerrin Palazzi, Christopher Oldmeadow, Constance Dimity Pond, Andrew Searles, Rod Ling, Remia Bruce, Wendy Murdoch, John Attia

**Affiliations:** 1 School of Nursing and Midwifery, College of Health, Medicine and Wellbeing, University of Newcastle, University Drive, Callaghan, NSW 2308, Australia; 2 Hunter New England Local Health District, Rankin Park, Newcastle, NSW 2287, Australia; s.prosserfenn@gmail.com (S.F.); anne.fullerton@health.nsw.gov.au (A.F.); carolyn.hullick@health.nsw.gov.au (C.H.); remia.bruce@health.nsw.gov.au (R.B.); wendy.murdoch@health.nsw.gov.au (W.M.); john.attia@newcastle.edu.au (J.A.); 3 Hunter Medical Research Institute, New Lambton Heights, Newcastle, NSW 2305, Australia; kerrin.palazzi@hmri.org.au (K.P.); christopher.oldmeadow@hmri.org.au (C.O.); andrew.searles@hmri.org.au (A.S.); rod.ling@hmri.org.au (R.L.); 4 School of Medicine and Public Health, College of Health, Medicine and Wellbeing, University of Newcastle, University Drive, Callaghan, NSW 2308, Australia; constance.pond2@gmail.com

**Keywords:** dementia, medications, polypharmacy, anticholinergic burden, medication reconciliation, acute care

## Abstract

People with dementia (PWD) are at risk for medication-related harm due to their impaired cognition and frequently being prescribed many medications. This study evaluated a medication safety intervention (including pharmacist medication reconciliation and review) for PWD during an unplanned admission to hospital. This article reports the effect of the intervention on polypharmacy, potentially inappropriate medications (PIMs), and anticholinergic burden scores for PWD. A pre-post design using an intervention site and a control site was conducted in 2017–2019, in a regional area in New South Wales, Australia. Polypharmacy, PIMs, and anticholinergic burden were measured at admission, discharge, and three months after discharge. There were 628 participants including 289 at the control site and 339 at the intervention site. Polypharmacy was 95% at admission and 90% at discharge. PIMs at admission were 95–98% across timepoints and decreased significantly at discharge. The mean anticholinergic score decreased significantly between admission (2.40–3.15) and discharge (2.01–2.57). Reduced PIMs at discharge were correlated with reduced anticholinergic burden (rho = 0.48–0.55, *p* < 0.001). No significant differences were identified between the study and control sites for Polypharmacy, PIMs, and anticholinergic burden rates and scores. High rates of polypharmacy and PIMs in this study indicate a study population with multiple comorbidities. This intervention was feasible to implement but was limited due to difficulty recruiting participants and deaths during the study. Future multisite studies should be designed to recruit larger study samples to evaluate interventions for improving medication safety for PWD and improve outcomes for these vulnerable people.

## 1. Introduction

People with dementia (PWD) have a high risk for adverse health outcomes associated with medications due to being cognitively impaired [1]. Cognitive impairment can contribute to missing medications due to confusion or memory problems or taking incorrect medications or dosages. The risk increases for PWD who do not have a carer, have multiple comorbidities and consequently have more than five medications prescribed for them (polypharmacy) [2,3], and who are prescribed potentially inappropriate medications (PIMs) [4]. PIMs are defined as “medications that pose potential risks that outweigh potential benefits” [5]. Many PIMs pose a risk for PWD because they have side effects that exacerbate confusion and balance problems (resulting in falls) and increase anticholinergic burden.

The prevalence of PIMs for PWD in the community has been reported in a systematic review to range from 10% to 56% and is higher in nursing home settings [5]. However, the prevalence of PIMs in hospital has been reported in another systematic review to be 53–90% for inpatients with cognitive impairment [6]. These reviews provide evidence to confirm that PIMs are an important clinical issue that requires further attention.

PWD admitted to hospital may also suffer escalated behavioural and psychological symptoms of dementia (BPSD) and may be temporarily managed with PIMs such as psychotropics, sedatives, or hypnotics. Antipsychotics have been identified as being overused and having limited clinical benefit for BPSD [7,8] and should not be continued for more than three months [9]. Consequently, the risk for PWD remains high if these medications are not discontinued at discharge [10].

Two previous studies have compared PIMs (using Revised Beers Criteria [11]) between admission and discharge and reported that PIMs for PWD were significantly reduced at discharge (mean 4.0 reduced to 3.3, difference 0.7, *p* < 0.0001), in a study of 277 admissions [1], and (mean reduced from 0.8 to 0.4, difference 0.4, *p* = 0.01) in a study of 118 admissions [12]. During hospitalisation, there is an opportunity to undertake medication review and reconciliation and to identify PIMs and other medications that may no longer be required. Where it is possible to reduce medications, the risk for PWD is also reduced. Some previous intervention studies have been conducted to reduce PIMs in community/primary care settings [13,14,15] and nursing homes, for older people [16]; however, no studies have been conducted in hospital settings on PWD for this purpose.

**Figure 1:**
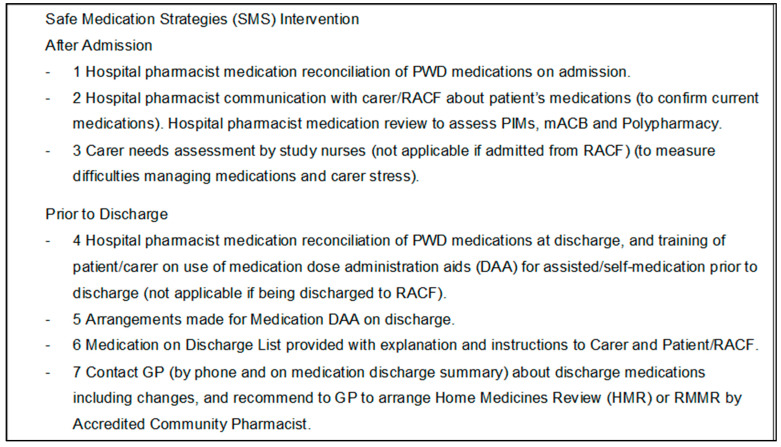
The Intervention—7 Safe Medication Strategies.

Previous studies of older people in non-hospital settings used interventions such as education interventions, medication reviews, and collaborative care approaches. Significant reductions in PIMs were reported from using medication reviews and an educational intervention. In addition, a prospective observational study of 991 pharmacist interventions for 557 patients during medication reconciliation in an emergency department reported that medication errors were severe in 57% of cases, and that 65% of the interventions were relevant [17]. This suggests that pharmacist medication review and reconciliation can be used to reduce PIMs and polypharmacy, as well as to reduce medication errors. Furthermore, a study that evaluated a collaborative care approach involving clinical pharmacist medication review, in which the clinical pharmacist was based in the community health centre with the general practitioner (GP), reported that 48% of recommendations were accepted by GPs in an elderly community population (*n* = 91) [14]. These previous studies describe approaches that may be relevant to PWD in hospital. Consequently, it is important to undertake research to evaluate the effectiveness of pharmacist interventions for PWD in the acute care setting. Interventions should be designed to reduce polypharmacy and PIMs (and associated anticholinergic burden) and measure the frequency of medication prescribing errors for these vulnerable admitted patients. The objective of this study was to evaluate an intervention designed to improve medication safety for PWD and their carers during an unplanned admission to hospital. The intervention was an evidence-based bundle of care including pharmacist medication reconciliation and review and is described in detail in Figure 1. The effects of the intervention on the primary outcomes are reported separately [18].

## 2. Materials and Methods

### 2.1. Study Design

This article reports the effect of the intervention on medication safety (secondary) outcomes of this study, including: polypharmacy, PIMS, and anticholinergic burden scores for PWD. In addition, we report the impact of the intervention on the frequency of medication review and reconciliation at admission and subsequent medication recommendations for PWD. A pre-post design was used because participants could not be randomised at the study sites due to the potential for inpatient transfers and staff mobility. The study was conducted at two public regional hospitals in New South Wales, Australia, between October 2017 and September 2019. There were two phases in this study, and usual care was delivered during phase one at both hospitals. In phase two, the intervention was delivered at the intervention hospital and the other hospital was used as a control site.

### 2.2. Study Participants

Participants were PWD or older people who had a history of dementia or a positive screen (Mini Mental State Examination (MMSE) score < 24/30 in the emergency department) for memory problems or confusion (excluding transient delirium), during an index admission via the emergency department (ED) during the study period. Proxy consent was provided by their carer or person responsible after the study was explained to them and they were provided with an information statement to consider. Patients were included in the study if they were admitted from home or a Residential Aged Care Facility (RACF), and each participant was only recruited into the study once. Additional detail about participant eligibility is provided in a separate publication [18].

### 2.3. Data Collection

Data were collected by a study pharmacist using study-specific clinical audit instruments for admission (usually within 48 h) and discharge (usually within 24 h), and medications from medical records to measure PIMs, numbers of medications, and to calculate the anticholinergic burden scores, using a modified anticholinergic burden score (mACB) (AUS) (based on the Anticholinergic Cognitive Burden Scale [19] and the Scottish NHS mARS Scale (https://www.sehd.scot.nhs.uk/publications/dc20150415polypharmacy.pdf, (accessed 28 August 2023) [20]) and modified for medications approved and in current use in Australia (see Figure S1 for modifications used for this study [19,20]) at admission and discharge. All medications were counted on admission and discharge including medications initiated on admission (within 24 h), separating combination medicines, over-the-counter medicines, complementary medicines, and when-required medicines. All routes (oral, inhaled, dermal, intravenous, etc.) of medicines were also included if prescribed in the timeframes.

Phone surveys of community pharmacists were conducted at three months after discharge to measure PIMs, numbers of medications, and to calculate the anticholinergic burden scores. Specific questions about medications were used to ensure these phone surveys were conducted in a standardized format.

In addition, severity and impact scores of prescribed medications were measured using the scoring system by Overhage and Lukes, as reported by Perez-Moreno et al. [17] to evaluate the potential impact of the prescribing error and the effect on the patient’s health. Errors were scored at admission by independent clinical pharmacists using five categories of severity ranging from no error to potentially lethal. Clinical relevance of the pharmacist recommendations (impact) was also scored using six likely consequences in patient care, ranging from injurious to extremely significant.

General practitioner acceptance of pharmacist recommendations following home medication review at three months after discharge was also measured using a post-discharge phone call by the study pharmacist to GPs.

### 2.4. The Intervention

The intervention was an evidence-based bundle of care [18] and comprised seven strategies delivered after admission and prior to discharge by the study pharmacist (see Figure 1). In this study, the delivery of medication review and reconciliation incorporated the following activities.

Medication reconciliation and medication review are distinct but interrelated processes. Medication reconciliation is a formal process of obtaining, verifying, and documenting an accurate list of a patient’s current medications on admission and comparing this list to the admission, transfer, and/or discharge medication orders to identify and resolve discrepancies. At the end of the episode of care, the verified information is transferred to the patient and next care provider. Medication reconciliation will often result in identification of medication discrepancies (e.g., differences between the documented medication history and the admission medication orders), which may be intentional or unintentional [21]. Medication review involves an evaluation of a patient’s medicines with the aim of optimising the quality use of medicines. A medication review will often result in the identification of actual or potential medication-related problems including identifying potentially inappropriate medications and polypharmacy and recommendations to optimise medicine use. After medication reconciliation was completed by the study pharmacist, a medication review was undertaken.

Usual care may have involved the delivery of some of these strategies individually but not as a routinely delivered bundle of care. Sample size calculations were based on the primary aim (treatment effect for readmissions/re-presentation to ED) and determined that 640 participants would be required. The evaluations of medication safety within the study reported in this paper are secondary outcomes; consequently, sample size calculations were not performed for these outcomes. Discharge and three-month data collection was not performed if consent was withdrawn, or the participant died prior to that timepoint.

### 2.5. Statistical Analysis

Polypharmacy was defined as ≥5 medications (including PRN medications) [22]. Scoring of medications using the mACB is listed in Figure S1, and the mean and median values were calculated for each phase and study site. Medication recommendation severity scores were dichotomised as “Significant (severity)” Yes (1, 2, 3) and No (4, 5), and also as “Due to error” (1, 2, 3, 4) and “Not due to error” (5); impact scores were dichotomised as “Relevant (impact)” Yes (1, 2, 3) and No (4, 5, 6), which is the approach used in a previous study [17]. As two pharmacists scored the recommendations, a sub-sample of recommendations was scored by both pharmacists (*n* = 59) and the agreement between pharmacists was tested using a weighted Kappa test.

Descriptive statistics were summarised by phase and site using means (SD) and median (min, max) for continuous data, and counts and percentages for categorical data. The change in medication use and medication review and reconciliation were examined from admission to discharge, and from discharge to 3 months across sites and phases using mixed modelling (negative binomial for count outcomes, logistic for dichotomous outcomes, and linear mixed modelling for normally distributed continuous outcomes); mixed modelling was used to account for correlations within a person over time. Fixed effects included phase, site, time (categorical), all two-way interactions, and a 3-way interaction term (phase*site*time), and (given adequate response numbers) included PWD characteristics identified as being potentially unbalanced between the sites (age, gender, discharge destination). A random effect was included for participant. The correlations between change in number of PIMs and mACB score (admission to discharge and discharge to 3 months) were examined (averaged over site and phase) using Spearman correlation; a non-parametric correlation test was used due to the large variation of the difference measure of both number of PIMs and mACB.

The treatment effect for mean count of medication recommendations (per participant) at admission was analysed using negative binomial regression. A zero-inflated negative binomial regression model was used to examine the treatment effect for number of “Significant (severity)” medication recommendations, medication recommendations “Not due to error”, and “Relevant (impact)” medication recommendations. A zero-inflation was added due to the increase in “0” counts from two “causes”—for example, 0 was used to represent that no medication recommendation was made, and also that no significant medication recommendation was made; the odds of having at least one medication recommendation was modelled together with the average count of “Significant (severity)” medication recommendations in participants who had at least one medication recommendation. Modelling included phase, site, and the interaction term (phase*site), and adjusted modelling included age, gender, and number of medications at admission.

The proportion of medication recommendations classed as “Significant (severity)”, as “Not due to error”, and as “Relevant (impact)” at admission was compared across phase and site using logistic mixed modelling; modelling included phase, site, and the interaction term (phase*site), and adjusted modelling included age, gender, and number of medications at admission. A random effect was included for participant to account for correlations within a person over multiple medication recommendations.

Data entry was undertaken by trained study team members using a data entry manual under the supervision of a senior researcher. Study data were collected and managed using REDCap (Research Electronic Data Capture) tools [23] hosted at the Hunter Medical Research Institute, Australia. Data were analysed using SAS v9.4 (SAS Institute Inc., Cary, NC, USA); a priori, *p* < 0.05 (two-tailed) was used to indicate statistical significance.

### 2.6. Ethics Approval and Consent to Participate

This study was conducted in accordance with the Declaration of Helsinki and was approved by the Hunter New England Health Human Research Ethics Committee (HREC) (17/06/21/4.08) and University of Newcastle (Australia) HREC (H-2017-0260). All participants had written consent provided by their carer or person responsible prior to their participation in the study.

## 3. Results

The final sample comprised 278 participants for the pre-intervention and 350 participants for the post-intervention phase. Patient characteristics by site and phase are presented in Table 1. There were some differences in gender (higher % of males at the control site), age (older mean age at the intervention site), and indigenous admissions (higher at the control site) between study sites and phases. For admissions from home, only 66% were discharged to home (343/523). Discharges to RACF increased by 69% overall compared with admissions from RACF (105 vs. 178), and 32 (5.1%) died during the index admission.

### 3.1. Medications, Polypharmacy, PIMs, and mACB Scores

Descriptive data for medications are presented in Table 2 by phase, site, and timepoint.

Polypharmacy was high overall, with an average of 95% at admission, decreasing to 90% at discharge. Adjusting for age, gender, and discharge destination, there were no significant differences in these proportions across sites and phases for both time points (admission to discharge *p* = 0.282, discharge to 3 months *p* = 0.894). See Supplementary Tables S1 and S2.

Over 95% of participants were prescribed PIMs across timepoints. All sites showed a significant decrease in the mean number of PIMs from admission to discharge. After adjusting for age, gender, and discharge destination, there was no significant treatment effect for PIMs at admission compared to discharge (*p* = 0.366), or at discharge compared to three months (*p* = 0.391). See Supplementary Tables S3 and S4.

The mean mACB score decreased for all phase/site combinations from admission to discharge; however, no treatment effect was seen (*p* = 0.086). The mean mACB score increased from discharge to 3 months at the control site in both phases, and did not change significantly at the intervention site, and no overall treatment effect was seen (*p* = 0.608). See Supplementary Tables S5 and S6.

Averaged over site and phase, significant moderate positive correlations were seen between PIMs change and mACB change from admission to discharge (rho = 0.48, *p* < 0.001), and from discharge to three months (rho = 0.55, *p* < 0.001). This is a clinically plausible and clinically significant correlation because reducing PIMs reduces anticholinergic burden.

### 3.2. PIMs Psychotropic and Sedative/Hypnotic Medication Categories

Psychotropic and Sedative/Hypnotic PIMs categories are shown in Table 3 by site and phase, and timepoint.

There were no differences in the proportion of participants on at least one psychotropic medication between sites and phases at each timepoint (admission to discharge *p* = 0.275, discharge to three months *p* = 0.915). See Supplementary Tables S7 and S8.

From admission to discharge, there was a significant decrease in the proportion of participants on at least one sedative/hypnotic medication at the control site in both phases and the intervention site in phase two; however, it could not be shown that this was due to the intervention (admission to discharge *p* = 0.233, discharge to three months *p* = 0.807). See Supplementary Tables S9 and S10.

### 3.3. Pharmacist Medication Review and Reconciliation Conducted at Admission and Prior to Discharge as Part of the Intervention

Pharmacist medication review and reconciliation conducted at admission and prior to discharge is shown in Table 4 by site, phase, and timepoint.

The increase in the proportion of participants receiving pharmacist medication review and reconciliation at admission was clinically significant in the Intervention group between phase one and phase two, while the control group remained stable (numbers too low to support regression modelling); similar was seen at discharge. A clinically significant result indicates that it is beneficial to patients’ quality of life even though it is not statistically significant [24].

### 3.4. Pharmacist Scoring of Prescribed Medications with Potential for Harm or Adverse Reaction or Prescribing Error per Participant at Admission

Pharmacists’ recommendations for medications that were identified during medication reconciliation as having a potential for harm or adverse reaction or prescribing error, were evaluated for their severity and relevance (impact of the service provided by the pharmacist).

Agreement between the two study pharmacists was found to be Almost Perfect (weighted Kappa 0.904, 95% CI 0.83–0.98). It was therefore determined to be appropriate to use scoring from both pharmacists for all subsequent analyses. Severity and impact scores for pharmacist’s medication recommendations (per participant) are shown in Table 5.

The proportion of participants with at least one medication recommendation at admission increased significantly in the Intervention group (OR 78.9, *p* < 0.001) and did not change at the control site, (OR 1.12, *p* = 0.676), and the overall effect was significant (OR 70.5, *p* < 0.001). The mean count of medication recommendations per participant increased significantly in the Intervention group between phase one and phase two (IRR 13.6, *p* < 0.001), while the control group remained stable (IRR 0.98, *p* = 0.923), with a significant overall treatment effect seen (IRR 13.8, *p* < 0.001). See Supplementary Tables S11 and S12.

Medication recommendations per participant scored as “Significant (severity)” was modelled as a two-part model; the increase (phase two compared to phase one) in the proportion of participants having at least one “Significant (severity)” medication recommendation was significantly higher at the intervention site than the control site (OR 20.5, *p* < 0.001). While a significant treatment effect was seen for the mean count of “Significant (severity)” medication recommendations (IRR 3.18, *p* < 0.001), with a significant increase in the intervention site from phase one to phase two (IRR 1.9, *p* = 0.022), and significant decrease in the control site (IRR 0.6, *p* = 0.006), the mean count at the intervention site in phase two was still lower than the mean count at the control site in phase one. See Supplementary Tables S13 and S14.

In addition, a treatment effect was seen in the proportion of the participants with at least one medication recommendation that was “Not due to error” (OR 65.2, *p* < 0.001), with a significant increase at the intervention site (OR 104, *p* < 0.001) but not the control site (OR 1.6, *p* = 0.18). See Supplementary Table S15.

The mean count of medication recommendations per participant scored as “Relevant (impact)” was modelled as a two-part model; the increase in the proportion of participants having at least one “Relevant (impact)” medication recommendation was significantly more at the intervention site for phase two compared to phase one, than the control site (OR 63.1, *p* < 0.001). A significant treatment effect was seen for the mean count per participant of “Relevant (impact)” medication recommendations (IRR 4.4, *p* < 0.001), with the count increasing significantly at the intervention site from phase one to phase two (IRR 4.5, *p* < 0.001), but not at the control site (IRR 1.01, *p* = 0.949). See Supplementary Tables S16 and S17.

### 3.5. Pharmacist Scoring of Prescribed Medications with Potential for Harm or Adverse Reaction or Prescribing Error (Recommendation Level) at Admission

Descriptive scores for medication recommendations (all medication recommendations, *n* = 1789) are shown in Table 6 by site and phase.

The proportion of medication recommendations that are “Significant (severity)” decreased significantly at the intervention site between phase one and phase two (OR 0.37, *p* = 0.006), although this decrease was not significantly different to the decrease seen at the control site (treatment effect OR 0.63, *p* = 0.328). See Supplementary Table S18.

There was a significant increase in the proportion of medication recommendations that did not involve a prescribing error at both sites in phase two, compared to phase one (Intervention OR 3, *p* = 0.003, Control OR 2.4, *p* = 0.008); however, the overall treatment effect was not significant (OR 1.2, *p* = 0.666). See Supplementary Table S19.

There was a significant increase in the proportion of relevant (impact of pharmacist service) recommendations at the intervention site in phase two, compared to phase one (OR 2.2, *p* = 0.031), although this change was not significantly different to the increase seen at the control site (treatment effect OR 1.2, *p* = 0.695). See Supplementary Table S20.

A significant moderate positive correlation was seen between severity and impact of medication recommendations at admission (*n* = 1446, Rho = 0.58, *p* < 0.001). This is a clinically plausible and clinically significant correlation because significant (severity) medication recommendations would be most likely to have a substantial impact on patient medication safety and quality of life.

### 3.6. GP Acceptance of Community Pharmacist Recommendations at Three Months

The GP acceptance of community pharmacist’s recommendations at three months after discharge was 68% (*n* = 104/156 recommendations).

In summary, there were no significant differences (no treatment effect) across sites and phases for polypharmacy, PIMs, and mACB scores. Clinically significant results include a significant moderate correlation between PIMS change and mACB scores, and an increase in the proportion of participants receiving pharmacist medication review and reconciliation at the intervention site, with a significant overall treatment effect in the mean count of medication recommendations. A treatment effect was also seen in the proportion of participants with at least one medication recommendation that was “not due to error”, and the mean count per participant of “relevant (impact)” medication recommendations, and a significant positive correlation was seen between severity and impact of medication recommendations at admission.

## 4. Discussion

The objective of this study was to evaluate an intervention designed to improve medication safety for PWD and their carers during an unplanned admission to hospital, and this paper reports the effect of the intervention on polypharmacy, PIMs, and anticholinergic burden.

Polypharmacy was high overall (>90%) and this reflects high comorbidity in this study population, and increasing comorbidity has been reported to be significantly associated with higher polypharmacy [25]. This result is higher than the rate reported in a study of older people including participants with cognitive impairment (*n* = 373) with a rate of 69% for polypharmacy [25] and a study that included (*n* = 10,528) participants with dementia in primary care of 57% [3]. These results indicate that polypharmacy is lower in the community for PWD.

Nonetheless, reduced prescribing reduces medication-related risk for PWD [1,25,26].

PIMs prescribing was very high (>90%) overall in this study. PWD who suffer escalated BPSD during a hospital admission may be temporarily managed with PIMs such as psychotropics, sedatives, or hypnotics. Previous studies have reported lower rates of PIMs prescribing (of at least one PIM) for PWD in the community. A multi-country study (*n* = 2004) reported a rate of 60% [4] and a nationwide study in New Zealand (*n* = 2190) reported a rate of 67% [27]. However, a recent systematic review reported that prevalence of PIMS ranged from 53–90% for inpatients with cognitive impairment [6], indicating that PIMs are likely to be higher in inpatient PWD. The mean number of PIMs decreased significantly (by 25%) from admission to discharge, and this also reduced medication-related risk for PWD [1,4]; however, no treatment effect was identified.

The mean mACB score decreased significantly from admission to discharge, and this suggests reduced risk for PWD; however, no treatment effect was identified. The significant moderate positive correlations between PIMs reduction and mACB reduction also indicate reduced medication-related risk for PWD in this study.

Prescribed psychotropic medications did not vary significantly from admission; however, 44% of patients were prescribed these medications. A report from Alzheimer’s Australia states that up to 20% of PWD who receive antipsychotic medications derive benefit from them [28], so there is potential for inappropriate prescribing for PWD in this study.

The proportion of participants on at least one sedative/hypnotic was significantly reduced at discharge at both sites (by 60%)—this is a clinically significant improvement in prescribing because it reduces risk for PWD, but no treatment effect was identified. This reduction may have been influenced by a recent initiative by the Australian Commission on Safety and Quality in Health Care that published National Safety and Quality Health Service Standards and targeted inappropriate prescribing for BPSD [7].

Medication review and reconciliation at admission and discharge increased significantly in the intervention site in phase two in this study. Medication review has been reported to significantly improve the appropriateness of prescribing in aged care facilities [15,16] and primary health care [14].

There was a significant increase in the mean number of medication recommendations identified by pharmacists during medication reconciliation at the intervention site in phase two. There was a high proportion of participants in the intervention group who received at least one medication recommendation (93%), and this was higher than the proportions for usual care/phase one participant groups (16–35%). This result is higher than the rate (19%) reported in an observational study (*n* = 2984) of admissions to emergency department (ED) [17] and may indicate that PWD require more modifications of their medications.

There was a significant increase in the proportion of participants having at least one “Significant (severity)” medication recommendation for the intervention group (72%) compared with the usual care groups (21%). Increased severity scores for the intervention group may have patient safety implications or may be the result of increased medication reconciliation at admission. The observational ED study reported a rate of 57% of significant severity recommendations [17]; however, medication reconciliation may not have been routinely conducted in this study.

The proportion of patients having at least one medication recommendation not due to error increased significantly at the intervention group (85%) compared with the usual care/phase one groups (5–20%). This suggests that pharmacists may have recommended modifications in medications rather than flagging potential drug interactions or adverse effects.

There was a significant increase in the proportion of participants having at least one relevant pharmacist medication recommendation in the intervention group (90%), indicating a clinically significant impact of the service provided by the pharmacist. The usual care/phase one rates ranged from 11–33%, and these were lower than the rate reported in the previous ED study (65%) [17], possibly due to inconsistent medication reconciliation. The significant moderate positive correlation between severity and impact scores in this study was similar to the correlation reported in the previous ED study (rho = 0.73, *p* < 0.001) [17].

The significant severity scores (by number of medication recommendations) were significantly lower at the intervention site in phase two compared with the control site in phase one. However, there was variation between the sites in phase one and between the phases at the control site, and this finding should be interpreted and extrapolated cautiously.

The proportion of medication recommendations that were not due to error (by number of medication recommendations) increased significantly at the intervention site between phase one and two; however, no treatment effect was identified.

There was a significant increase in the relevance (impact) of medication recommendations (by number of medication recommendations) at the intervention site between phase one and two; however, no treatment effect was identified.

GP acceptance of at least one HMR recommendation at three months after discharge was 68%, and this was higher than an observational study in primary care (*n* = 91) in which GP acceptance of 304/625 pharmacist recommendations (48%) was reported [14], and a study (*n* = 1021) reporting GP acceptance of ED pharmacist recommendations (49%) [29]. The reason for this difference is unknown.

Previous studies have reported the effect of interventions on medication safety for older people in the community or primary care settings [13,14,15,16]; however, this study evaluated the effectiveness of a pharmacist intervention for PWD inpatients and the effect on polypharmacy and PIMS. Although this study did not find a treatment effect for the intervention, the clinically significant results have implications for future research. Medication safety for PWD is particularly important because of the risks associated with medications for PWD. The Australian Health Ministers Advisory Council has established nine National Health Priority Areas, including dementia, and in 2019 they announced that quality use of medicines and medicine safety will be the 10th National Health Priority Area in Australia [30]. The addition of this priority area should emphasize the importance of this issue for future research and practice improvement initiatives.

### 4.1. Clinical Implications

PWD or cognitive impairment are not always identified at admission. Consequently, clinicians may not recognise that this vulnerable group of patients needs particular attention regarding their medications. This is not a trivial issue. If routine screening is conducted in emergency departments to identify older people with cognitive impairment, then clinicians can recognize and respond by paying careful attention to escalating BPSD and associated medication issues. PWD often have polypharmacy and many associated medication safety concerns. Polypharmacy may be a consequence of their complex comorbidities. Pharmacist-led medication review and reconciliation is a valuable means of ensuring medication safety for PWD and can result in them having improved outcomes due to reductions in polypharmacy, PIMs, and deprescribing.

### 4.2. Strengths and Limitations

Few previous studies have been conducted exclusively on PWD in acute care and focused on their medication safety. This is significant because many studies specifically exclude PWD due to the difficulty associated with their recruitment and participation in studies due to their cognitive impairment. The inclusion of carers in this study to provide proxy consent for participants and to confirm current medications at admission was a strength of this study. PWD or cognitive impairment is not always identified at admission. Having an intervention that focuses on PWD in an inpatient setting requires robust systems for identification of PWD that are sensitive to their needs and values. This study undertook screening to identify PWD at admission and evaluated the effect of a pharmacist-led intervention on polypharmacy and PIMs for PWD in acute care. The study design only used two sites and participants were not randomised, and this limits the internal validity and generalisability of the results. There was also potential for selection bias and confounding factors in this study, and outcomes assessors were not blinded. The study was limited due to difficulty recruiting participants and 5% deaths during admission resulting in a smaller study population than specified in the sample size calculation, and this may have resulted in not being able to demonstrate a treatment effect. In addition, values of all regression findings should be interpreted cautiously due to low numbers and only two study sites. The STROBE Statement guidelines for reporting observational studies was used to review and prepare this manuscript [31].

## 5. Conclusions

This study found a clinically significant reduction in medication-related risks for PWD admitted to hospital. Admission to hospital offers a pivotal moment to undertake medication reconciliation, thereby minimizing medication-related risks, reducing polypharmacy and PIMs, identifying potential drug interactions, side effects, dosage modifications, and deprescribing. Given the complex medication regimens and the associated vulnerabilities of PWD, optimizing their medication during hospital admission can significantly impact their overall health outcomes and quality of life. This study highlights the need for focused medication management in this high-risk population when they are admitted to hospital compared with studies conducted in the community. The intervention was feasible to implement because it used existing clinical strategies and combined them into a routinely delivered bundle of care. This intervention may be used to inform future multisite studies that would have sufficient power to demonstrate treatment effect and improve medication safety and quality of life for PWD in acute care. Given the evident benefits and feasibility, it is imperative for healthcare systems to prioritize routine screening for cognitive impairment in older people at admission and to conduct medication reconciliation processes for PWD, setting the stage for safer and more patient-centred care.

## Figures and Tables

**Table 1:** Patient characteristics, by phase and site.

		Phase 1		Phase 2		
Characteristic	Class/Statistic	Control (*n* = 117)	Intervention (*n* = 161)	Control (*n* = 172)	Intervention (*n* = 178)	Total (*n* = 628)
Gender	Male	66 (56%)	73 (45%)	98 (57%)	73 (41%)	310 (49%)
	Female	51 (44%)	88 (55%)	74 (43%)	105 (59%)	318 (51%)
Age	Mean (SD)	83 (7)	86 (7)	82 (8)	84 (8)	84 (8)
Aboriginal and/or TSI	Yes	2 (1.7%)	1 (0.6%)	10 (5.8%)	3 (1.7%)	16 (2.6%)
	Missing	1	0	0	1	2
CCI	Mean (SD)	2 (2)	2 (2)	2 (2)	2 (2)	2 (2)
CCI group	0	23 (20%)	33 (21%)	35 (20%)	49 (28%)	140 (22%)
	1–2	62 (53%)	89 (56%)	87 (51%)	89 (51%)	327 (52%)
	3–4	22 (19%)	22 (14%)	34 (20%)	26 (15%)	104 (17%)
	5+	10 (8.5%)	16 (10%)	15 (8.8%)	12 (6.8%)	53 (8.5%)
	Missing	0	1	1	2	4
Admitted from	Home	97 (83%)	125 (78%)	149 (87%)	152 (85%)	523 (83%)
	RACF	20 (17%)	36 (22%)	23 (13%)	26 (15%)	105 (17%)
Discharge	Home (GP)	68 (59%)	80 (50%)	98 (57%)	100 (56%)	346 (55%)
	RACF	27 (23%)	57 (36%)	42 (24%)	52 (29%)	178 (29%)
	Transitions program	1 (0.9%)	2 (1.3%)	3 (1.7%)		6 (1.0%)
	Transfer to other Acute Care Facility	15 (13%)	14 (8.8%)	14 (8.1%)	6 (3.4%)	49 (7.9%)
	Rehabilitation Facility			7 (4.1%)	4 (2.2%)	11 (1.8%)
	Died during index admission	3 (2.6%)	6 (3.8%)	8 (4.7%)	15 (8.5%)	32 (5.1%)
	Missing	3	2	0	1	6

CCI: Charlson Comorbidity Index; RACF: Residential Aged Care Facility; TSI: Torres Strait Islander.

**Table 2:** Medications by phase and site, and timepoint.

			Phase 1		Phase 2		
Characteristic	Timepoint	Class/Statistic	Control (*n* = 117)	Intervention (*n* = 161)	Control (*n* = 172)	Intervention (*n* = 178)	Total (*n* = 628)
Number of medications	Admission	Mean (SD)	12 (5)	11 (4)	14 (5)	13 (5)	13 (5)
		Median (min, max)	12 (2, 27)	11 (1, 21)	13 (1, 28)	14 (1, 33)	12 (1, 33)
		Missing	1	0	0	0	1
	Discharge	Mean (SD)	10 (4)	9 (4)	10 (4)	9 (4)	9 (4)
		Median (min, max)	10 (2, 20)	8 (1, 20)	10 (2, 25)	9 (1, 28)	9 (1, 28)
		Missing	10	10	21	20	61
	3 month	Mean (SD)	11 (4)	9 (4)	10 (4)	9 (4)	9 (4)
		Median (min, max)	10 (2, 22)	9 (2, 20)	9 (2, 21)	8 (1, 28)	9 (1, 28)
		Missing	31	50	43	49	173
Polypharmacy	Admission	Yes	110 (95%)	150 (93%)	169 (98%)	168 (94%)	597 (95%)
		Missing	1	0	0	0	1
	Discharge	Yes	99 (93%)	133 (88%)	139 (92%)	141 (89%)	512 (90%)
		Missing	10	10	21	20	61
	3 month	Yes	83 (97%)	103 (93%)	117 (91%)	111 (86%)	414 (91%)
		Missing	31	50	43	49	173
PIMs prescribed	Admission	Yes	116 (100%)	154 (96%)	171 (99%)	176 (99%)	617 (98%)
		Missing	1	0	0	0	1
	Discharge	Yes	106 (99%)	138 (91%)	148 (98%)	148 (94%)	540 (95%)
		Missing	10	10	21	20	61
	3 month	Yes	85 (99%)	107 (96%)	124 (96%)	118 (91%)	434 (95%)
		Missing	31	50	43	49	173
Number of PIMs	Admission	Mean (SD)	4 (2)	4 (2)	5 (2)	4 (2)	4 (2)
		Median (min, max)	4 (1, 10)	3 (0, 11)	5 (0, 17)	4 (0, 11)	4 (0, 17)
		Missing	1	0	0	0	1
	Discharge	Mean (SD)	4 (2)	3 (2)	4 (2)	3 (2)	3 (2)
		Median (min, max)	4 (0, 9)	3 (0, 8)	4 (0, 10)	3 (0, 8)	3 (0, 10)
		Missing	10	10	21	20	61
	3 month	Mean (SD)	4 (2)	3 (2)	4 (2)	3 (2)	4 (2)
		Median (min, max)	4 (0, 10)	3 (0, 8)	4 (0, 9)	3 (0, 9)	3 (0, 10)
		Missing	31	50	43	49	173
MACB total	Admission	Mean (SD)	3 (2)	2 (2)	3 (2)	3 (2)	3 (2)
		Median (min, max)	3 (0, 9)	2 (0, 15)	3 (0, 15)	3 (0, 10)	2 (0, 15)
		Missing	1	0	0	0	1
	Discharge	Mean (SD)	3 (2)	2 (2)	3 (2)	2 (2)	2 (2)
		Median (min, max)	2 (0, 10)	2 (0, 10)	2 (0, 9)	2 (0, 8)	2 (0, 10)
		Missing	10	10	21	20	61
	3 month	Mean (SD)	3 (2)	2 (2)	3 (2)	2 (1)	2 (2)
		Median (min, max)	3 (0, 10)	2 (0, 7)	2 (0, 10)	2 (0, 5)	2 (0, 10)
		Missing	31	50	43	49	173

MACB: modified Anticholinergic Burden.

**Table 3:** Psychotropic and Sedative/hypnotic PIMS, by phase and site, and timepoint.

			Phase 1		Phase 2		
Characteristic	Timepoint	Class/Statistic	Control (*n* = 117)	Intervention (*n* = 161)	Control (*n* = 172)	Intervention (*n* = 178)	Total (*n* = 628)
Psychotropic medication	Admission	No	63 (54%)	102 (63%)	99 (58%)	90 (51%)	354 (56%)
		Yes	53 (46%)	59 (37%)	73 (42%)	88 (49%)	273 (44%)
		Missing	1	0	0	0	1
	Discharge	No	60 (56%)	92 (61%)	86 (57%)	89 (56%)	327 (58%)
		Yes	47 (44%)	59 (39%)	65 (43%)	69 (44%)	240 (42%)
		Missing	10	10	21	20	61
	3 month	No	44 (51%)	62 (56%)	68 (53%)	70 (54%)	244 (54%)
		Yes	42 (49%)	49 (44%)	61 (47%)	59 (46%)	211 (46%)
		Missing	31	50	43	49	173
Sedative/hypnotic medication	Admission	No	97 (84%)	141 (88%)	140 (81%)	148 (83%)	526 (84%)
		Yes	19 (16%)	20 (12%)	32 (19%)	30 (17%)	101 (16%)
		Missing	1	0	0	0	1
	Discharge	No	99 (93%)	140 (93%)	139 (92%)	153 (97%)	531 (94%)
		Yes	8 (7.5%)	11 (7.3%)	12 (7.9%)	5 (3.2%)	36 (6.3%)
		Missing	10	10	21	20	61
	3 month	No	77 (90%)	95 (86%)	116 (90%)	122 (95%)	410 (90%)
		Yes	9 (10%)	16 (14%)	13 (10%)	7 (5.4%)	45 (9.9%)
		Missing	31	50	43	49	173

**Table 4:** Pharmacist medication review and reconciliation, by phase and site, and timepoint.

			Phase 1		Phase 2		
Characteristic	Timepoint	Class/Statistic	Control (*n* = 117)	Intervention (*n* = 161)	Control (*n* = 172)	Intervention (*n* = 178)	Total (*n* = 628)
Medication reconciliation	Admission	No	75 (65%)	111 (69%)	101 (59%)	1 (0.6%)	288 (46%)
		Yes	41 (35%)	49 (31%)	71 (41%)	173 (97%)	334 (53%)
		Unable to deliver				4 (2.2%)	4 (0.6%)
		Missing	1	1	0	0	2
Medication reconciliation	Discharge	No	109 (99.1%)	111 (74%)	156 (95%)	4 (2.5%)	380 (65%)
		Yes	1 (0.9%)	38 (26%)	8 (4.9%)	139 (85%)	186 (32%)
		Unable to deliver				20 (12%)	20 (3.4%)
		Missing	7	12	8	15	42

**Table 5:** Medication recommendations (admission, per participant), by phase and site.

		Phase 1		Phase 2		
Characteristic	Class/Statistic	Control (*n* = 117)	Intervention (*n* = 161)	Control (*n* = 172)	Intervention (*n* = 178)	Total (*n* = 628)
At least 1 medication recommendation	No	82 (70%)	135 (84%)	111 (65%)	12 (6.7%)	340 (54%)
	Yes	35 (30%)	26 (16%)	61 (35%)	166 (93%)	288 (46%)
Number of recommendations	mean (SD)	1 (2)	0 (1)	1 (2)	6 (4)	2 (3)
	Median (min, max)	0 (0, 10)	0 (0, 11)	0 (0, 10)	6 (0, 26)	0 (0, 26)
At least 1 Significant (Severity) medication recommendation	No	88 (75%)	143 (89%)	122 (71%)	49 (28%)	402 (64%)
	Yes	29 (25%)	18 (11%)	50 (29%)	129 (72%)	226 (36%)
Number of (Significant Severity) recommendations	mean (SD)	1 (1)	0 (1)	1 (1)	2 (2)	1 (1)
	Median (min, max)	0 (0, 6)	0 (0, 4)	0 (0, 6)	2 (0, 8)	0 (0, 8)
At least 1 medication recommendation—not due to error (severity 5)	No	103 (88%)	153 (95%)	138 (80%)	27 (15%)	421 (67%)
	Yes	14 (12%)	8 (5.0%)	34 (20%)	151 (85%)	207 (33%)
Number of medications not due to error	mean (SD)	0 (1)	0 (1)	0 (1)	3 (3)	1 (2)
	Median (min, max)	0 (0, 5)	0 (0, 11)	0 (0, 9)	3 (0, 23)	0 (0, 23)
At least 1 Relevant (Impact) medication recommendation	No	87 (74%)	143 (89%)	116 (67%)	18 (10%)	364 (58%)
	Yes	30 (26%)	18 (11%)	56 (33%)	160 (90%)	264 (42%)
Number of Relevant (Impact) recommendations	mean (SD)	1 (1)	0 (1)	1 (2)	4 (3)	2 (3)
	Median (min, max)	0 (0, 5)	0 (0, 4)	0 (0, 9)	4 (0, 24)	0 (0, 24)

**Table 6:** Medication recommendations (admission, recommendation level), by phase and site.

		Phase 1		Phase 2		
Characteristic	Class/Statistic	Control (*n* = 211)	Intervention (*n* = 196)	Control (*n* = 316)	Intervention (*n* = 1066)	Total (*n* = 1789)
Significant (Severity) medication recommendation	No	46 (36%)	29 (49%)	102 (50%)	725 (69%)	902 (62%)
	Yes	83 (64%)	30 (51%)	103 (50%)	329 (31%)	545 (38%)
	Missing	82	137	111	12	342
Severity of the prescription error	2. Serious	10 (7.8%)	10 (17%)	12 (5.9%)	51 (4.8%)	83 (5.7%)
	3. Significant	73 (57%)	20 (34%)	91 (44%)	278 (26%)	462 (32%)
	4. Least	19 (15%)	7 (12%)	20 (9.8%)	104 (9.9%)	150 (10%)
	5. No error	27 (21%)	22 (37%)	82 (40%)	621 (59%)	752 (52%)
	Missing	82	137	111	12	342
Relevant (Impact) medication recommendation	No	56 (43%)	32 (52%)	63 (31%)	324 (31%)	475 (33%)
	Yes	73 (57%)	29 (48%)	142 (69%)	730 (69%)	974 (67%)
	Missing	82	135	111	12	340
Impact of the service provided by the pharmacist	1. Extremely significant				3 (0.3%)	3 (0.2%)
	2. Highly significant	5 (3.9%)	11 (18%)	11 (5.4%)	99 (9.4%)	126 (8.7%)
	3. Significant	68 (53%)	18 (30%)	131 (64%)	628 (60%)	845 (58%)
	4. Little significant	15 (12%)	6 (9.8%)	10 (4.9%)	35 (3.3%)	66 (4.6%)
	5. Insignificant	40 (31%)	26 (43%)	53 (26%)	289 (27%)	408 (28%)
	6. Injurious Intervention	1 (0.8%)				1 (0.1%)
	Missing	82	135	111	12	340

## Data Availability

Data available on request due to ethical restrictions, because the investigators do not have permission from participants to make the data available; however, the study team would be pleased to be contacted by any research groups that may be interested in collaborating on future work.

## Supplementary Materials

The following supporting information can be downloaded at: https://www.mdpi.com/article/10.3390/healthcare11202771/s1, Figure S1: Modified Anticholinergic Burden (mACB) (AUS) [19,20]; Tables S1–S20: Supplementary Tables presenting results of statistical modelling.

## Abbreviations

SMS: Safe Medication Strategy; PWD: People with dementia; PIM: Potentially inappropriate medication; RACF: Residential aged care facility; ED: Emergency department; mACB (AUS): modified Anticholinergic Cognitive Burden Scale (Australia); HREC: Human Research Ethics Committee; BPSD: Behavioural and Psychological Symptoms of Dementia; GP: General Practitioner.
