# Supplementary material for: Evaluation of the Effect of a Safe Medication Strategy on Potentially Inappropriate Medications, Polypharmacy and Anticholinergic Burden for People with Dementia: An Intervention Study
Source: Healthcare (Basel). 2023 Oct 19;11(20):2771. doi: 10.3390/healthcare11202771 (PMC10606387; doi:10.3390/healthcare11202771)
Supplement: Supplementary file 1 [file healthcare-11-02771-s001.zip › healthcare-2610929-supplementary (Figures).pdf]

Figure S1: MODIFIED ANTICHOLINERGIC BURDEN (mACB) (AUS)

DRUG (ALPHABETICAL ORDER)	ANTICHOLINERGIC BURDEN SCORE
Alprazolam	1
Amantadine	2
Amitriptyline	3
Aripiprazole	1
Asenapine	1
Atenolol	1
Atropine	3
Baclofen	2
Belladonna	2
Benzatropine	3
Brompheniramine	3
Bupropion	1
Captopril	1
Carbamazepine	2
Cetirizine	2
Chlorpheniramine	3
Chlorpromazine	3
Chlorthalidone	1
Cimetidine	2
Clomipramine	3
Clozapine	3
Codeine	1
Colchicine	1
Cyproheptadine	1

Darifenacin	3
Desipramine	2
Desloratadine	1
Diazepam	1
Digoxin	1
Diphenhydramine	3
Dipyridamole	1
Disopyramide	1
Doxepin	3
Doxylamine	3
Fentanyl	1
Fluphenazine	1
Fluvoxamine	1
Furosemide (frusemide)	1
Haloperidol	1
Hydralazine	1
Hydrocortisone	1
Hyoscyamine	3
Imipramine	3
Isosorbide	1
Levocetirizine	1
Loperamide	1
Loratadine	2
Metoprolol	1
Metoclopramide	1
Mirtazapine	1
Morphine	1
Nifedipine	1

Nortriptyline	3
Olanzapine	3
Orphenadrine	3
Oxybutynin	3
Oxcarbazepine	2
Paliperidone	1
Paroxetine	3
Periciazine	2
Prednisone/prednisolone	1
Prochlorperazine	2
Promethazine	3
Propantheline	3
Pseudoephedrine	2
Quetiapine	3
Quinidine	1
Ranitidine	1
Reboxetine	1
Risperidone	1
Sertraline	2
Solifenacin	3
Theophylline	3
Thioridazine	3
Tiotropium	2
Tolterodine	3
Triamterene	1
Trifluoperazine	3
Trihexyphenidyl (benzhexol)	3

Trimipramine	3
Venlafaxine	1
Warfarin	1

References:

Polypharmacy Guidance Scottish NHS Trust United Kingdom using the Anticholinergic Risk Scale (ARS) ranking medication with anticholinergic potential on a scale of: 1. Moderate, 2. Strong, 3. Very strong. [1]

Anticholinergic Cognitive Burden Scale 2012 Developed by the Aging Brain Program of the Indiana Center for Aging Research [2] ACB score of:
1. Evidence from in vitro data that chemical entity has antagonist activity at receptor,
2. Evidence from literature, prescribers information and clinical effect,
3. Evidence from literature, expert opinion and prescribers information that medication may cause delirium

NOTE: The following approach was used to modify this ACB Score:
- When a drug had different scores in the two references used, the higher score was assigned.
- When a drug was only listed in one reference then the score for that reference was used.
- The modifications in this scale include only medications approved and in current use in Australia.

1. Scottish Government Model of Care Polypharmacy Working Group. Polypharmacy Guidance **2015**. https://www.sehd.scot.nhs.uk/publications/dc20150415polypharmacy.pdf,

2. Aging Brain Program. Anticholinergic Cognitive Burden Scale. **2012**. https://www.idhca.org/wp-content/uploads/2018/02/DESAI_ACB_scale_-_Legal_size_paper.pdf
