# Supplementary material for: Evaluation of the Effect of a Safe Medication Strategy on Potentially Inappropriate Medications, Polypharmacy and Anticholinergic Burden for People with Dementia: An Intervention Study
Source: Healthcare (Basel). 2023 Oct 19;11(20):2771. doi: 10.3390/healthcare11202771 (PMC10606387; doi:10.3390/healthcare11202771)
Supplement: Supplementary file 1 [file healthcare-11-02771-s001.zip › healthcare-2610929-supplementary (Table).pdf]

Supplementary Tables

Table S1: *Polypharmacy at Admission compared to Discharge**

		Predicted proportion (95%CI)		*Change in proportion*		*Overall effect*
Phase	*Site*	*Admission*	*Discharge*	*OR (95%CI)***	*p-value*	*p-value*
Phase 1	Control	0.95 (0.89, 0.98)	0.93 (0.86, 0.97)	0.67 (0.22, 2.07)	0.489	0.282
	Intervention	0.94 (0.89, 0.97)	0.90 (0.83, 0.94)	0.53 (0.23, 1.19)	0.123	.
Phase 2	Control	0.98 (0.95, 1.00)	0.93 (0.87, 0.96)	0.20 (0.05, 0.74)	0.016	.
	Intervention	0.95 (0.91, 0.98)	0.91 (0.85, 0.94)	0.49 (0.21, 1.13)	0.094	.

*adjusted for age, gender, discharge destination **Discharge vs Admission

Table S2: *Polypharmacy at Discharge compared to 3 months**

		Predicted proportion (95%CI)		*Change in proportion*		*Overall effect*
Phase	*Site*	*Discharge*	*3 month*	*OR (95%CI)***	*p-value*	*p-value*
Phase 1	Control	0.94 (0.87, 0.97)	0.97 (0.91, 0.99)	2.45 (0.60, 10.0)	0.212	0.894
	Intervention	0.90 (0.84, 0.94)	0.94 (0.88, 0.97)	1.83 (0.73, 4.59)	0.201	.
Phase 2	Control	0.93 (0.88, 0.96)	0.92 (0.86, 0.96)	0.89 (0.37, 2.14)	0.791	.
	Intervention	0.92 (0.86, 0.95)	0.89 (0.82, 0.94)	0.76 (0.36, 1.62)	0.479	.

*adjusted for age, gender, discharge destination **3 month vs Discharge

Table S3: *PIMs at Admission compared to Discharge**

		Estimated mean count (95%CI)		*Change in count*		*Overall effect*
Phase	*Site*	*Admission*	*Discharge*	*IRR (95%CI)***	*p-value*	*p-value*
Phase 1	Control	4.27 (3.90, 4.68)	3.71 (3.38, 4.08)	0.87 (0.81, 0.93)	<.001	0.366
	Intervention	3.62 (3.32, 3.94)	2.83 (2.56, 3.14)	0.78 (0.73, 0.84)	<.001	.
Phase 2	Control	4.78 (4.47, 5.12)	3.69 (3.42, 3.98)	0.77 (0.73, 0.82)	<.001	.
	Intervention	4.31 (3.99, 4.65)	2.81 (2.56, 3.10)	0.65 (0.61, 0.70)	<.001	.

*adjusted for age, gender, discharge destination **Discharge vs Admission

Table S4: *PIMs at Discharge compared to 3 months**

Phase	Site	Estimated mean count (95%CI) Discharge	3 month	Change in count IRR (95%CI)**	p-value	Overall effect p-value
Phase 1	Control	3.73 (3.39, 4.10)	4.16 (3.77, 4.59)	1.11 (1.03, 1.21)	0.010	0.391
	Intervention	2.87 (2.59, 3.18)	3.31 (2.99, 3.66)	1.15 (1.07, 1.24)	<.001	.
Phase 2	Control	3.69 (3.42, 3.99)	3.81 (3.50, 4.15)	1.03 (0.97, 1.10)	0.334	.
	Intervention	2.86 (2.59, 3.15)	2.85 (2.55, 3.19)	1.00 (0.93, 1.07)	0.951	.

*adjusted for age, gender, discharge destination **3 month vs Discharge

Table S5: *Mean mACB score at Admission compared to Discharge**

Phase	Site	LS-Mean (95%CI) Admission	Discharge	Change in mean Difference (95%CI)**	p-value	Overall effect p-value
Phase 1	Control	3.02 (2.64, 3.41)	2.57 (2.19, 2.94)	-.46 (-.72, -.19)	<.001	0.086
	Intervention	2.40 (2.09, 2.70)	2.08 (1.77, 2.39)	-.32 (-.54, -.09)	0.005	.
Phase 2	Control	3.15 (2.78, 3.52)	2.47 (2.16, 2.78)	-.68 (-.94, -.42)	<.001	.
	Intervention	2.99 (2.64, 3.34)	2.01 (1.74, 2.29)	-.97 (-1.2, -.72)	<.001	.

*adjusted for age, gender, discharge destination **Discharge vs Admission

Table S6: *Mean mACB score at Discharge compared to 3 months**

Phase	Site	LS-Mean (95%CI) Discharge	3 month	Change in mean Difference (95%CI)**	p-value	Overall effect p-value
Phase 1	Control	2.65 (2.27, 3.03)	3.10 (2.68, 3.53)	0.45 (0.15, 0.75)	0.003	0.608
	Intervention	2.14 (1.82, 2.45)	2.33 (2.00, 2.66)	0.19 (-.06, 0.44)	0.140	.
Phase 2	Control	2.53 (2.20, 2.85)	2.76 (2.40, 3.11)	0.23 (0.04, 0.42)	0.016	.
	Intervention	2.09 (1.81, 2.37)	1.94 (1.68, 2.19)	-.15 (-.31, 0.01)	0.066	.

*adjusted for age, gender, discharge destination **3 month vs Discharge

Table S7: *Psychotropic medications at Admission compared to Discharge**

Phase	Site	Predicted proportion (95%CI) Admission	Discharge	Change in proportion OR (95%CI)**	p-value	Overall effect p-value
Phase 1	Control	0.45 (0.33, 0.58)	0.43 (0.30, 0.56)	0.90 (0.48, 1.70)	0.750	0.275
	Intervention	0.35 (0.25, 0.47)	0.39 (0.28, 0.50)	1.15 (0.66, 2.01)	0.620	.
Phase 2	Control	0.39 (0.29, 0.50)	0.39 (0.28, 0.50)	0.99 (0.58, 1.69)	0.965	.
	Intervention	0.52 (0.41, 0.62)	0.42 (0.31, 0.53)	0.67 (0.40, 1.13)	0.135	.

*adjusted for age, gender, discharge destination **Discharge vs Admission

Table S8: *Psychotropic medications at Discharge compared to 3 months**

Phase	Site	Predicted proportion (95%CI) Discharge	3 month	Change in proportion OR (95%CI)**	p-value	Overall effect p-value
Phase 1	Control	0.43 (0.31, 0.57)	0.51 (0.37, 0.66)	1.38 (0.70, 2.75)	0.352	0.915
	Intervention	0.39 (0.29, 0.51)	0.47 (0.35, 0.61)	1.39 (0.75, 2.56)	0.289	.
Phase 2	Control	0.41 (0.30, 0.52)	0.46 (0.34, 0.58)	1.25 (0.70, 2.24)	0.453	.
	Intervention	0.44 (0.34, 0.56)	0.48 (0.36, 0.61)	1.17 (0.66, 2.08)	0.580	.

*adjusted for age, gender, discharge destination **3 month vs Discharge

Table S9: *Sedative/Hypnotic medications at Admission compared to Discharge**

Phase	Site	Predicted proportion (95%CI) Admission	Discharge	Change in proportion OR (95%CI)**	p-value	Overall effect p-value
Phase 1	Control	0.15 (0.09, 0.24)	0.06 (0.03, 0.13)	0.39 (0.15, 0.96)	0.041	0.233
	Intervention	0.11 (0.07, 0.17)	0.06 (0.03, 0.11)	0.49 (0.22, 1.14)	0.097	.
Phase 2	Control	0.17 (0.11, 0.24)	0.06 (0.03, 0.12)	0.34 (0.16, 0.72)	0.005	.
	Intervention	0.16 (0.11, 0.23)	0.03 (0.01, 0.07)	0.15 (0.06, 0.41)	<.001	.

*adjusted for age, gender, discharge destination **Discharge vs Admission

Table S10: *Sedative/Hypnotic medications at Discharge compared to 3 months**

		Predicted proportion (95%CI)		*Change in proportion*		*Overall effect*
Phase	*Site*	*Discharge*	*3 month*	*OR (95%CI)***	*p-value*	*p-value*
Phase 1	Control	0.07 (0.03, 0.14)	0.11 (0.05, 0.21)	1.69 (0.59, 4.87)	0.329	0.807
	Intervention	0.06 (0.03, 0.11)	0.14 (0.08, 0.23)	2.79 (1.15, 6.77)	0.023	.
Phase 2	Control	0.07 (0.04, 0.12)	0.10 (0.05, 0.17)	1.53 (0.64, 3.67)	0.343	.
	Intervention	0.03 (0.01, 0.07)	0.06 (0.02, 0.12)	1.96 (0.58, 6.54)	0.276	.

*adjusted for age, gender, discharge destination **3 month vs Discharge

Table S11: *At least 1 medication recommendation (Admission)**

n = 627	*Site effect* Predicted probability (95%) Phase 1	*Phase 2*	*Phase effect* Odds ratio (95%CI)***	*p-value*	*Overall effect* Odds ratio (95%CI)****	*p-value*
Control	0.31 (0.23, 0.41)	0.34 (0.27, 0.42)	1.12 (0.66, 1.90)	0.676	70.5 (28.0, 177)	<.001
Intervention	0.16 (0.11, 0.22)	0.94 (0.89, 0.96)	78.9 (37.2, 168)	<.001		.
Odds Ratio (95%CI)**	0.40 (0.22, 0.74)	28.5 (14.3, 57.1)		.		.
p-value	0.003	<0.001		.		.

*adjusted for age, gender, number of medications **Intervention vs Control ***Phase 2 vs Phase 1
****Intervention change vs Control change

Table S12: *Medication recommendations (Admission) – participant level**

	Site effect		*Phase effect*		*Overall effect*	
	Phase 1 *Estimated Incidence Rate*	*Phase 2* *Estimated Incidence Rate*	*IRR (95%CI)****	*p-value*	*IRR (95%CI)*****	*p-value*
Control	1.07 (0.83, 1.38)	1.05 (0.85, 1.30)	0.98 (0.71, 1.36)	0.923	13.8 (8.65, 22.1)	<.001
Intervention	0.38 (0.28, 0.51)	5.19 (4.42, 6.09)	13.6 (9.70, 19.1)	<.001		.
IRR (95%CI)**	0.36 (0.24, 0.53)	4.93 (3.79, 6.41)		.		.
p-value	<0.001	<0.001		.		.

*adjusted for age, gender, number of medications **Intervention vs Control ***Phase 2 vs Phase 1
****Intervention change vs Control change

Table S13: *"Significant (severity)" medication recommendations (Admission, Yes/No)-participant level**

	Site effect Predicted probability (95%CI)		*Overall effect Odds ratio*	
Site	*Phase 1*	*Phase 2*	*(95%CI)***	*p-value*
Control	0.29 (0.20, 0.39)	0.37 (0.27, 0.48)	20.5 (6.43, 65.4)	<.001
Intervention	0.15 (0.08, 0.26)	0.84 (0.72, 0.92)		

*adjusted for age, gender, number of medications **Intervention change vs Control change

Table S14: *Count of "Significant (severity)" medication recommendations (Admission) in participants with at least 1 "Significant (severity)" medication recommendation**

	Site effect Estimated mean count (95%CI)		*Phase effect*		*Overall effect*	
	Phase 1	*Phase 2*	*IRR (95%CI)****	*p-value*	*IRR (95%CI)*****	*p-value*
Control	2.38 (1.82, 3.11)	1.43 (1.08, 1.88)	0.60 (0.42, 0.86)	0.006	3.18 (1.64, 6.16)	<.001
Intervention	1.04 (0.60, 1.80)	1.99 (1.70, 2.33)	1.91 (1.10, 3.31)	0.022		.
IRR (95%CI)**	0.44 (0.24, 0.79)	1.39 (1.04, 1.86)		.		.
p-value	0.007	0.024		.		.

*adjusted for age, gender, number of medications **Intervention vs Control ***Phase 2 vs Phase 1 ****Intervention change vs Control change

Table S15: *At least 1 medication recommendation not due to error (Admission)**

	Site effect Predicted probability (95%)		*Phase effect Odds ratio*		*Overall effect Odds ratio*	
n = 627	*Phase 1*	*Phase 2*	*(95%CI)****	*p-value*	*(95%CI)*****	*p-value*
Control	0.12 (0.07, 0.20)	0.18 (0.13, 0.25)	1.60 (0.80, 3.18)	0.180	65.2 (22.2, 192)	<.001
Intervention	0.05 (0.02, 0.10)	0.85 (0.78, 0.89)	104 (45.3, 240)	<.001		.
Odds Ratio (95%CI)**	0.38 (0.15, 0.96)	25.0 (13.9, 44.8)		.		.
p-value	0.04	<0.001		.		.

*adjusted for age, gender, number of medications **Intervention vs Control ***Phase 2 vs Phase 1 ****Intervention change vs Control change

Table S16: *”Relevant (impact)” medication recommendations (Admission, Yes/No)- participant level**

	Site effect *Predicted probability (95%CI)*		*Overall effect* *Odds ratio*	
	Phase 1	*Phase 2*	*(95%CI)***	*p-value*
Control	0.33 (0.23, 0.45)	0.39 (0.29, 0.49)	63.1 (15.7, 253)	<.001
Intervention	0.19 (0.10, 0.32)	0.95 (0.87, 0.98)		

*adjusted for age, gender, number of medications **Intervention change vs Control change

Table S17: *Count of ”Relevant (impact)” medication recommendations (Admission) in participants with at least 1 ”Relevant (impact)” medication recommendation**

	Site effect *Estimated mean count (95%CI)*		*Phase effect*		*Overall effect*	
	Phase 1	*Phase 2*	*IRR (95%CI)****	*p-value*	*IRR (95%CI)*****	*p-value*
Control	1.85 (1.34, 2.55)	1.87 (1.48, 2.37)	1.01 (0.68, 1.50)	0.949	4.43 (2.17, 9.03)	<.001
Intervention	0.88 (0.48, 1.59)	3.94 (3.52, 4.41)	4.48 (2.46, 8.15)	<.001		.
IRR (95%CI)**	0.48 (0.24, 0.93)	2.10 (1.64, 2.70)		.		.
p-value	0.03	<0.001		.		.

*adjusted for age, gender, number of medications **Intervention vs Control ***Phase 2 vs Phase 1 ****Intervention change vs Control change

Table S18: *”Significant (severity)” medication recommendations (Admission)- recommendation level**

	Site effect *Predicted probability (95%)*		*Phase effect*		*Overall effect*	
	Phase 1	*Phase 2*	*Odds ratio (95%CI)****	*p-value*	*Odds ratio (95%CI)*****	*p-value*
Control	0.66 (0.54, 0.76)	0.54 (0.44, 0.63)	0.59 (0.32, 1.09)	0.094	0.63 (0.25, 1.60)	0.328
Intervention	0.54 (0.37, 0.70)	0.30 (0.26, 0.35)	0.37 (0.18, 0.75)	0.006		.
Odds Ratio (95%CI)**	0.60 (0.26, 1.39)	0.38 (0.25, 0.57)		.		.
p-value	0.231	<0.001		.		.

*adjusted for age, gender, number of medications **Intervention vs Control ***Phase 2 vs Phase 1 ****Intervention change vs Control change

Table S19: *Medication recommendations not due to error (Severity=5, Admission)-recommendation level**

	Site effect *Predicted probability (95%)*		*Phase effect* *Odds ratio*		*Overall effect* *Odds ratio*	
	Phase 1	*Phase 2*	*(95%CI)****	*p-value*	*(95%CI)*****	*p-value*
Control	0.19 (0.12, 0.28)	0.36 (0.28, 0.45)	2.43 (1.26, 4.69)	0.008	1.24 (0.47, 3.30)	0.666
Intervention	0.32 (0.19, 0.49)	0.59 (0.54, 0.64)	3.02 (1.46, 6.23)	0.003		.
Odds Ratio (95%CI)**	2.08 (0.85, 5.05)	2.57 (1.69, 3.91)		.		.
p-value	0.107	<0.001		.		.

*adjusted for age, gender, number of medications **Intervention vs Control ***Phase 2 vs Phase 1 ****Intervention change vs Control change

Table S20: *"Relevant (impact)" medication recommendations (Admission)- recommendation level**

	Site effect *Predicted probability (95%)*		*Phase effect* *Odds ratio*		*Overall effect* *Odds ratio*	
	Phase 1	*Phase 2*	*(95%CI)****	*p-value*	*(95%CI)*****	*p-value*
Control	0.59 (0.47, 0.70)	0.72 (0.63, 0.79)	1.79 (0.95, 3.36)	0.070	1.21 (0.47, 3.09)	0.695
Intervention	0.52 (0.36, 0.68)	0.70 (0.66, 0.74)	2.16 (1.07, 4.35)	0.031		.
Odds Ratio (95%CI)**	0.76 (0.33, 1.74)	0.91 (0.58, 1.44)		.		.
p-value	0.512	0.698		.		.

*adjusted for age, gender, number of medications **Intervention vs Control ***Phase 2 vs Phase 1 ****Intervention change vs Control change
